# Supplementary material for: Species distribution modeling for disease ecology: A multi-scale case study for schistosomiasis host snails in Brazil
Source: PLOS Glob Public Health. 2024 Aug 2;4(8):e0002224. doi: 10.1371/journal.pgph.0002224 (PMC11296653; doi:10.1371/journal.pgph.0002224)
Supplement: S1 Text — (DOCX) [file pgph.0002224.s001.docx]

**Species distribution modeling for disease ecology: a multi-scale case study for schistosomiasis host snails in Brazil**

# Supplemental Appendix

Table A: Mean and +/- standard errors of in-sample AUC, sensitivity, specificity, pAUC, pAUC significance, and TSS values for national models of all species across machine learning model types. Values are calculated across 10 bootstrapping iterations in which models were provided 80% of the available species presence records.

| **Species** | **Model Type** | **AUC** | **Sensitivity** | **Specificity** | **pAUC** | **pAUC**  **p-value** | **TSS** |
| --- | --- | --- | --- | --- | --- | --- | --- |
| *B. glabrata* | MaxEnt | 0.86  (0.86–0.86) | 0.87  (0.86–0.88) | 0.71  (0.70–0.73) | 0.74  (0.74–0.74) | 0.00  (0.00–0.00) | 0.58 (0.58–0.59) |
|  | RF | 0.99  (0.99–0.99) | 0.99  (0.99–0.99) | 0.99  (0.99–0.99) | 0.83  (0.83–0.83) | 0.00  (0.00–0.00) | 0.99  (0.99–0.99) |
|  | BRT | 0.98 (0.97–0.98) | 0.94 (0.93–0.95) | 0.90 (0.89–0.92) | 0.82  (0.81–0.82) | 0.00  (0.00–0.00) | 0.84  (0.83–0.86) |
| *B. straminea* | MaxEnt | 0.89 (0.88–0.89) | 0.84  (0.83–0.85) | 0.79 (0.78–0.80) | 0.76  (0.76–0.76) | 0.00  (0.00–0.00) | 0.63 (0.62–0.64) |
|  | RF | 0.99  (0.99–0.99) | 0.99  (0.99–0.99) | 0.99  (0.99–0.99) | 0.83  (0.83–0.83) | 0.00  (0.00–0.00) | 0.99 (0.99–0.99) |
|  | BRT | 0.98  (0.97–0.98) | 0.94 (0.94–0.95) | 0.91  (0.90–0.91) | 0.82  (0.82–0.82) | 0.00  (0.00–0.00) | 0.85 (0.84–0.86) |
| *B. tenagophila* | MaxEnt | 0.91  (0.91–0.92) | 0.86 (0.86–0.87) | 0.83 (0.83–0.84) | 0.78  (0.77–0.78) | 0.00  (0.00–0.00) | 0.69 (0.69–0.70) |
|  | RF | 0.91 (0.91–0.92) | 0.86 (0.86–0.87) | 0.83 (0.83–0.84) | 0.83  (0.77–0.78) | 0.00  (0.00–0.00) | 0.70 (0.69–0.70) |
|  | BRT | 0.97 (0.97–0.97) | 0.90 (0.89–0.92) | 0.90 (0.89–0.91) | 0.83  (0.83–0.83) | 0.00  (0.00–0.00) | 0.80 (0.80–0.81) |

Table B: Mean and +/- standard errors of in-sample AUC, sensitivity, specificity, pAUC, pAUC significance, and TSS values calculated on state-level presence and background records for nationally-fit and state-specific models. *B. glabrata* and *B. straminea* were tested in Minas Gerais state, and *B. tenagophila* was tested in São Paulo state. Values are calculated across 10 bootstrapping iterations in which models were provided 80% of the available species presence records.

| **Species** | **Model Type** | **Geographic extent** | **AUC** | **Sensitivity** | **Specificity** | **pAUC** | **pAUC**  **p-value** | **TSS** |
| --- | --- | --- | --- | --- | --- | --- | --- | --- |
| *B. glabrata* | MaxEnt | State | 0.77  (0.76–0.77) | 0.80  (0.79–0.82) | 0.63  (0.61–0.65) | 0.68  (0.67–0.68) | 0.00  (0.00–0.00) | 0.44  (0.42–0.45) |
|  |  | National cropped | 0.58  (0.58–0.60) | 0.83  (0.80–0.85) | 0.35  (0.32–0.38) | 0.56  (0.55–0.57) | 0.03  (0.02–0.04) | 0.18  (0.16–0.20) |
|  | RF | State | 0.99  (0.99–0.99) | 0.98  (0.98–0.99) | 0.97  (0.97–0.97) | 0.81  (0.81–0.82) | 0.00  (0.00–0.00) | 0.95  (0.95–0.96) |
|  |  | National cropped | 0.90  (0.90–0.90) | 0.85  (0.84–0.86) | 0.81  (0.81–0.82) | 0.77  (0.76–0.77) | 0.00  (0.00–0.00) | 0.66  (0.66–0.67) |
|  | BRT | State | 0.91  (0.90–0.92) | 0.85  (0.83–0.86) | 0.83  (0.82–0.85) | 0.78  (0.77–0.78) | 0.00  (0.00–0.00) | 0.68  (0.66–0.70) |
|  |  | National cropped | 0.81  (0.80–0.81) | 0.87  (0.86–0.88) | 0.63  (0.62–0.64) | 0.70  (0.70–0.71) | 0.00  (0.00–0.00) | 0.50  (0.49–0.50) |
| *B. straminea* | MaxEnt | State | 0.70  (0.70–0.72) | 0.71  (0.68–0.73) | 0.63  (0.62–0.65) | 0.64  (0.63–0.65) | 0.00  (0.00–0.00) | 0.34  (0.31–0.36) |
|  |  | National cropped | 0.65  (0.65–0.66) | 0.71  (0.69–0.73) | 0.58  (0.55–0.60) | 0.60  (0.60–0.61) | 0.00  (0.00–0.00) | 0.28  (0.28–0.29) |
|  | RF | State | 0.99  (0.99–0.99) | 0.98  (0.98–0.99) | 0.97  (0.97–0.98) | 0.82  (0.81–0.82) | 0.00  (0.00–0.00) | 0.96  (0.95–0.96) |
|  |  | National cropped | 0.90  (0.90–0.90) | 0.86  (0.85–0.87) | 0.82  (0.81–0.83) | 0.77  (0.76–0.77) | 0.00  (0.00–0.00) | 0.68  (0.68–0.69) |
|  | BRT | State | 0.88  (0.87–0.89) | 0.85  (0.83–0.87) | 0.77  (0.76–0.78) | 0.75  (0.74–0.76) | 0.00  (0.00–0.00) | 0.62  (0.60–0.64) |
|  |  | National cropped | 0.79  (0.79–0.80) | 0.83  (0.82–0.83) | 0.67  (0.66–0.68) | 0.69  (0.69–0.70) | 0.00  (0.00–0.00) | 0.50  (0.49–0.50) |
| *B. tenagophila* | MaxEnt | State | 0.85  (0.85–0.86) | 0.87  (0.86–0.88) | 0.72  (0.71–0.73) | 0.74  (0.73–0.74) | 0.00  (0.00–0.00) | 0.59  (0.59–0.60) |
|  |  | National cropped | 0.82  (0.82–0.82) | 0.80  (0.79–0.82) | 0.71  (0.69–0.72) | 0.71  (0.71–0.71) | 0.00  (0.00–0.00) | 0.51  (0.51–0.51) |
|  | RF | State | 0.99  (0.99–0.99) | 0.99  (0.99–0.99) | 0.99  (0.99–0.99) | 0.82  (0.82–0.83) | 0.00  (0.00–0.00) | 0.99  (0.99–0.99) |
|  |  | National cropped | 0.92  (0.91–0.92) | 0.89  (0.89–0.90) | 0.83  (0.82–0.83) | 0.78  (0.78–0.78) | 0.00  (0.00–0.00) | 0.72  (0.72–0.72) |
|  | BRT | State | 0.96  (0.95–0.96) | 0.93  (0.92–0.94) | 0.86  (0.86–0.87) | 0.81  (0.80–0.81) | 0.00  (0.00–0.00) | 0.79  (0.78–0.80) |
|  |  | National cropped | 0.88  (0.88–0.88) | 0.92  (0.91–0.92) | 0.73  (0.72–0.74) | 0.75  (0.75–0.75) | 0.00  (0.00–0.00) | 0.65  (0.64–0.65) |

Fig A: Maps of environmental covariates across geographic scales (national, Minas Gerais, and São Paulo state). Bioclimatic variables (i.e. temperature and precipitation) are colored in orange, soil-related variables in blue, and land-use/land-cover variables in green. Maps were built in R (version 4.2.2) using shapefiles from the *geobr* package [1].

1. Temperature seasonality (standard deviation of the monthly mean temperatures)
2. Mean daily temperature of the coldest quarter
3. Mean monthly precipitation of the wettest quarter
4. Mean monthly precipitation amount of the driest quarter
5. Soil clay percentage
6. Height above nearest drainage
7. Soil pH
8. Soil water percentage
9. Proportion of temporary crop cover
10. Distance to high population density


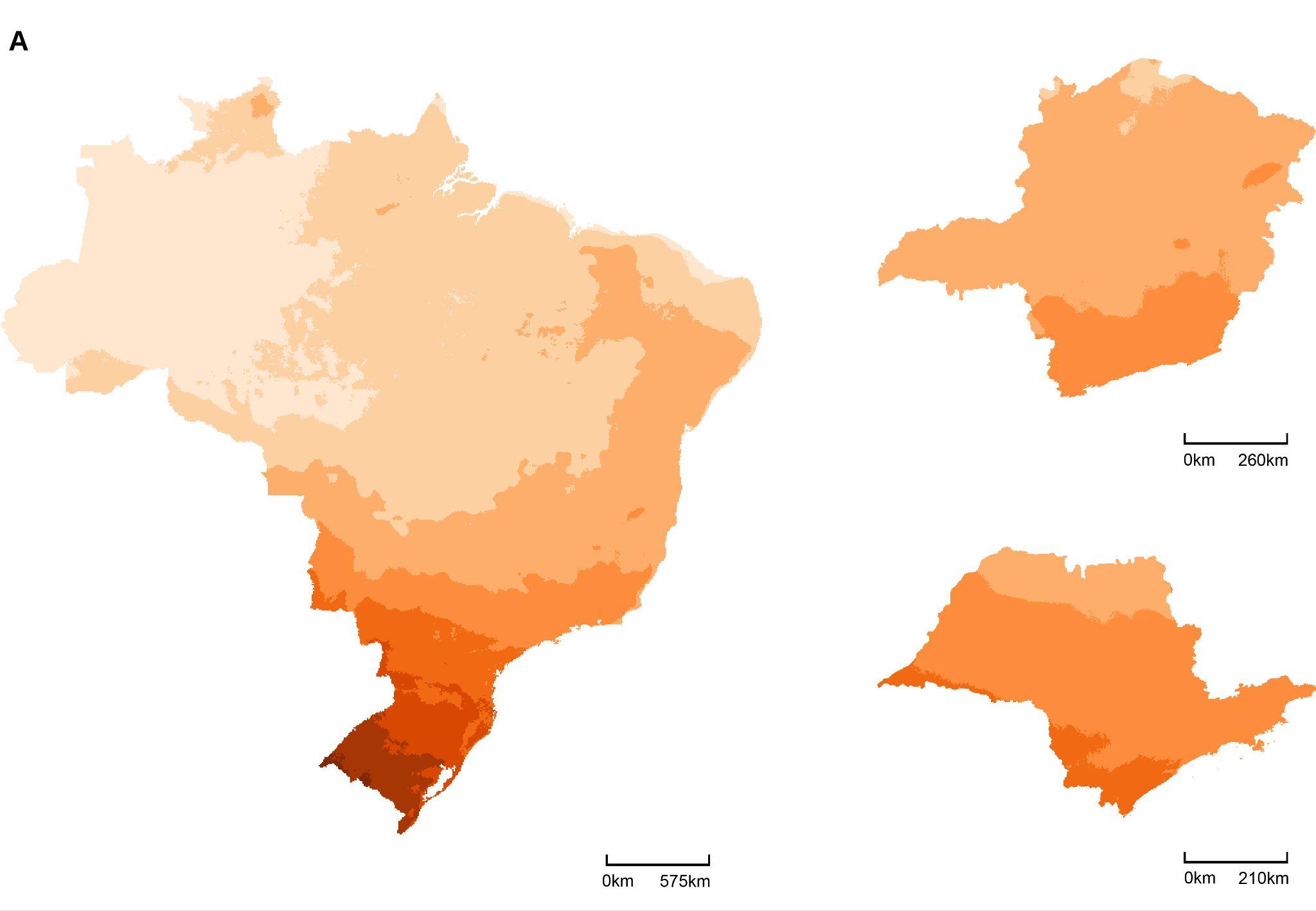

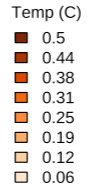


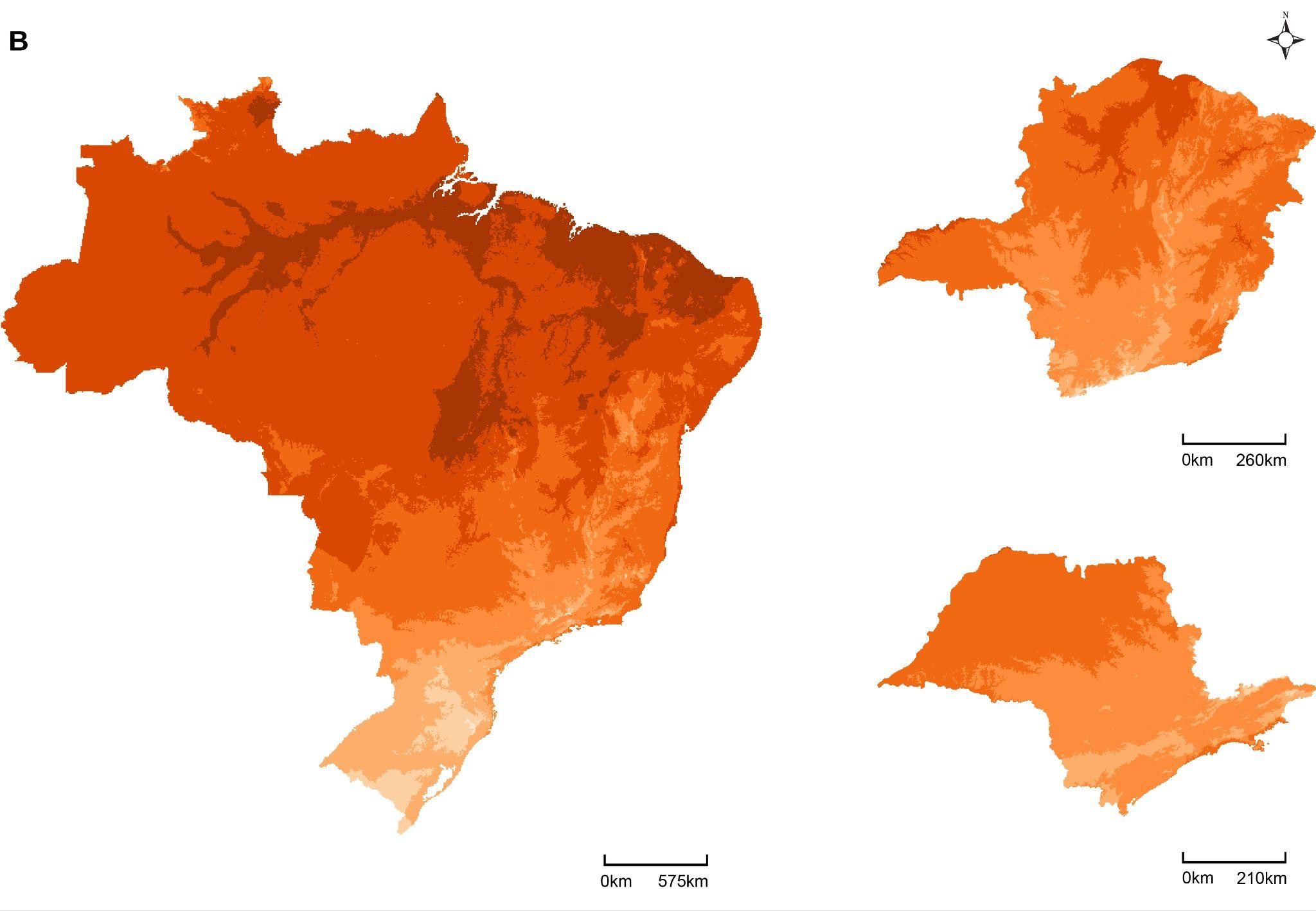

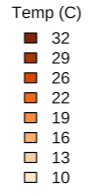


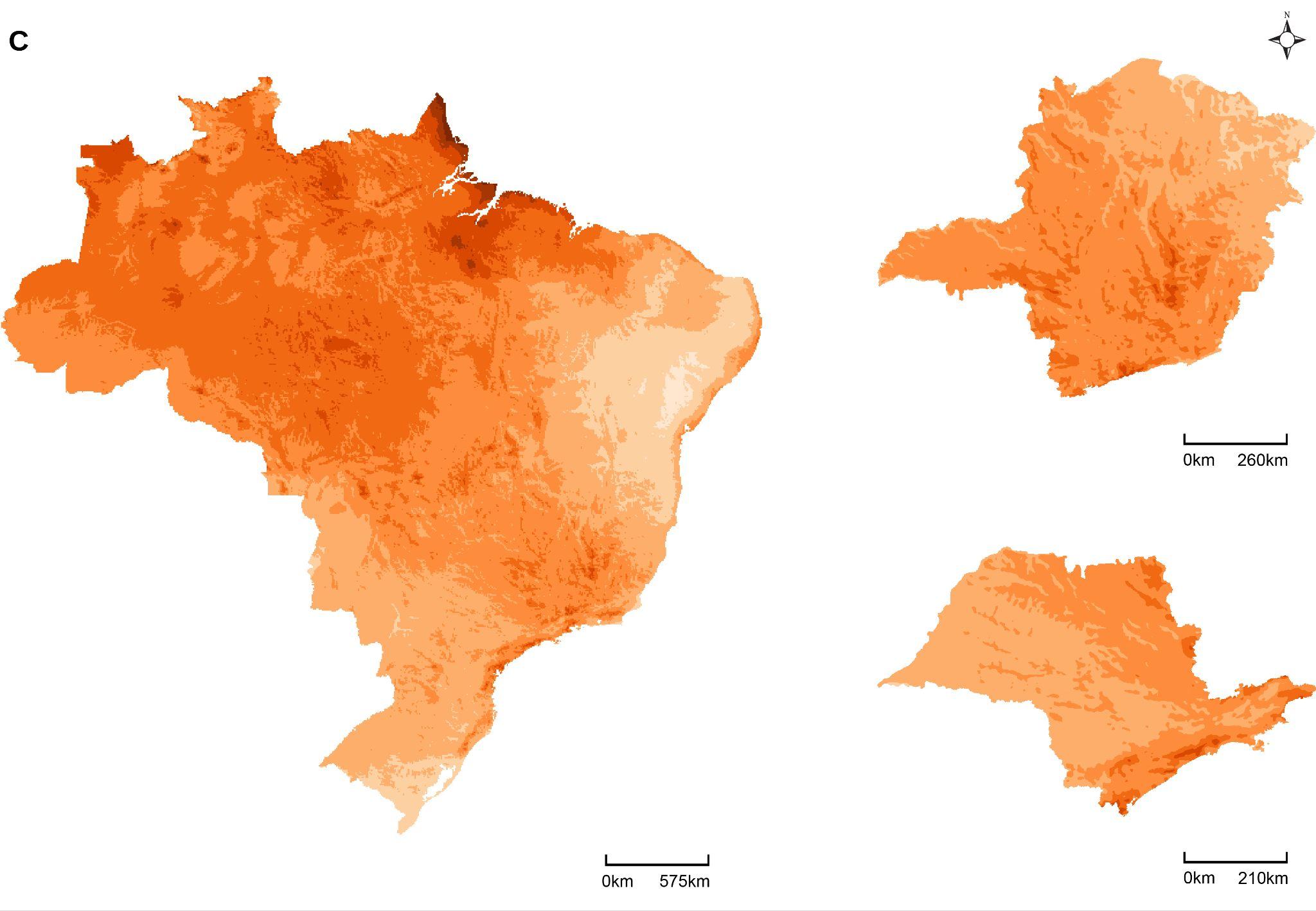

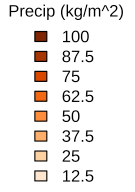


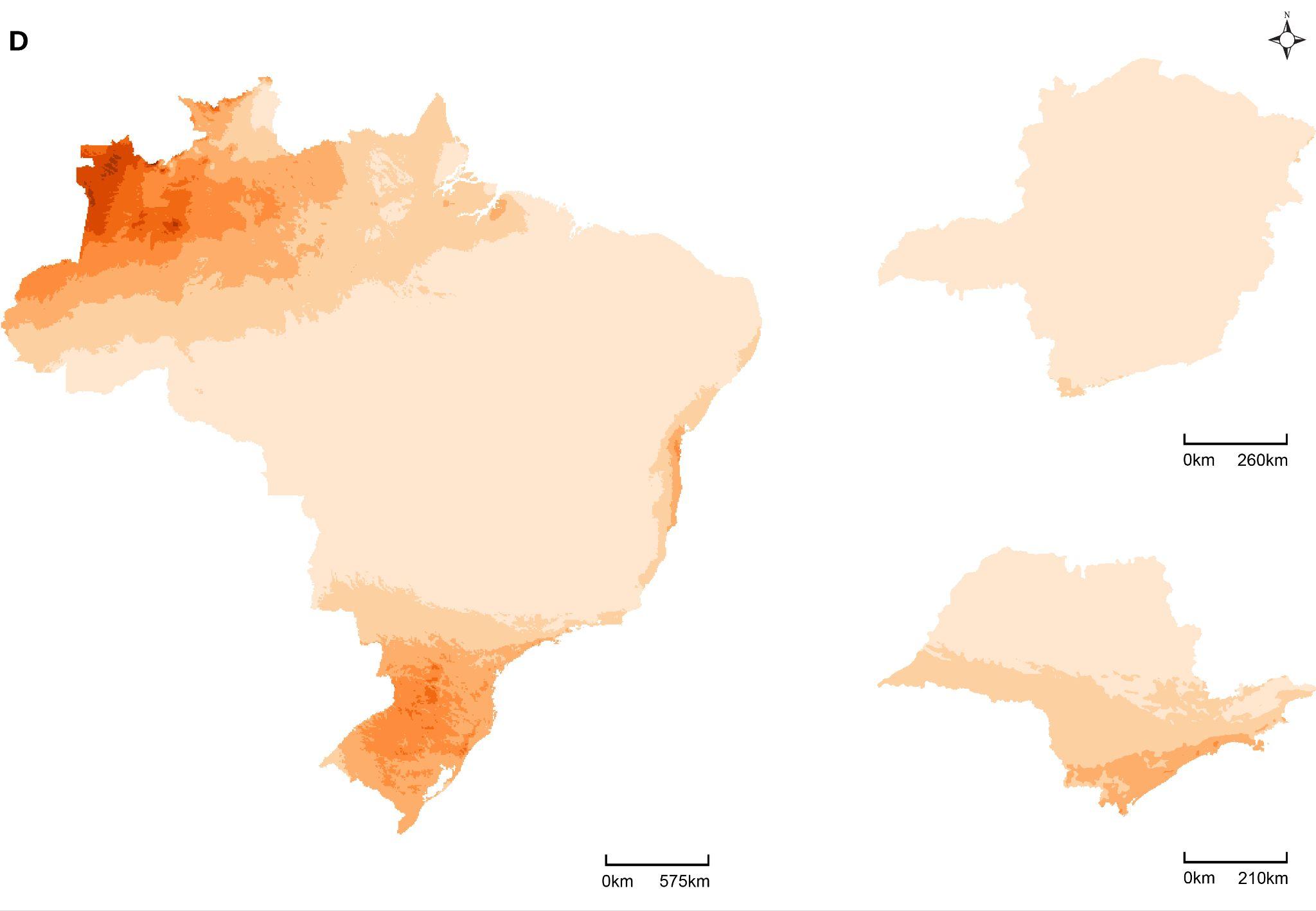

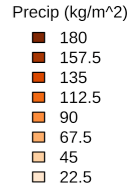


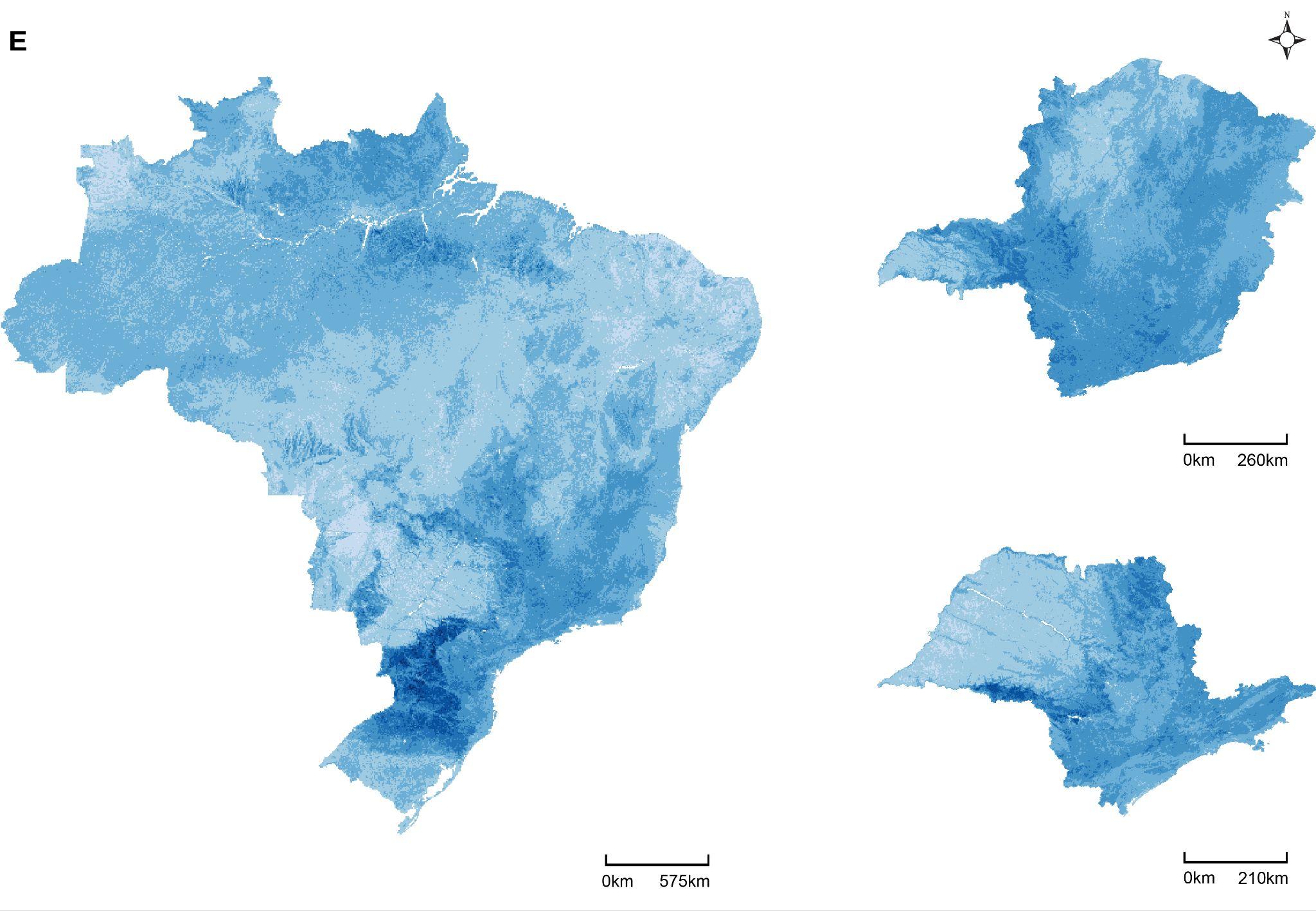

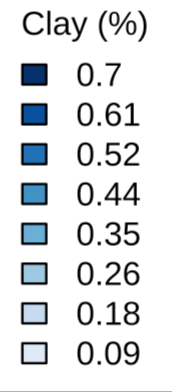


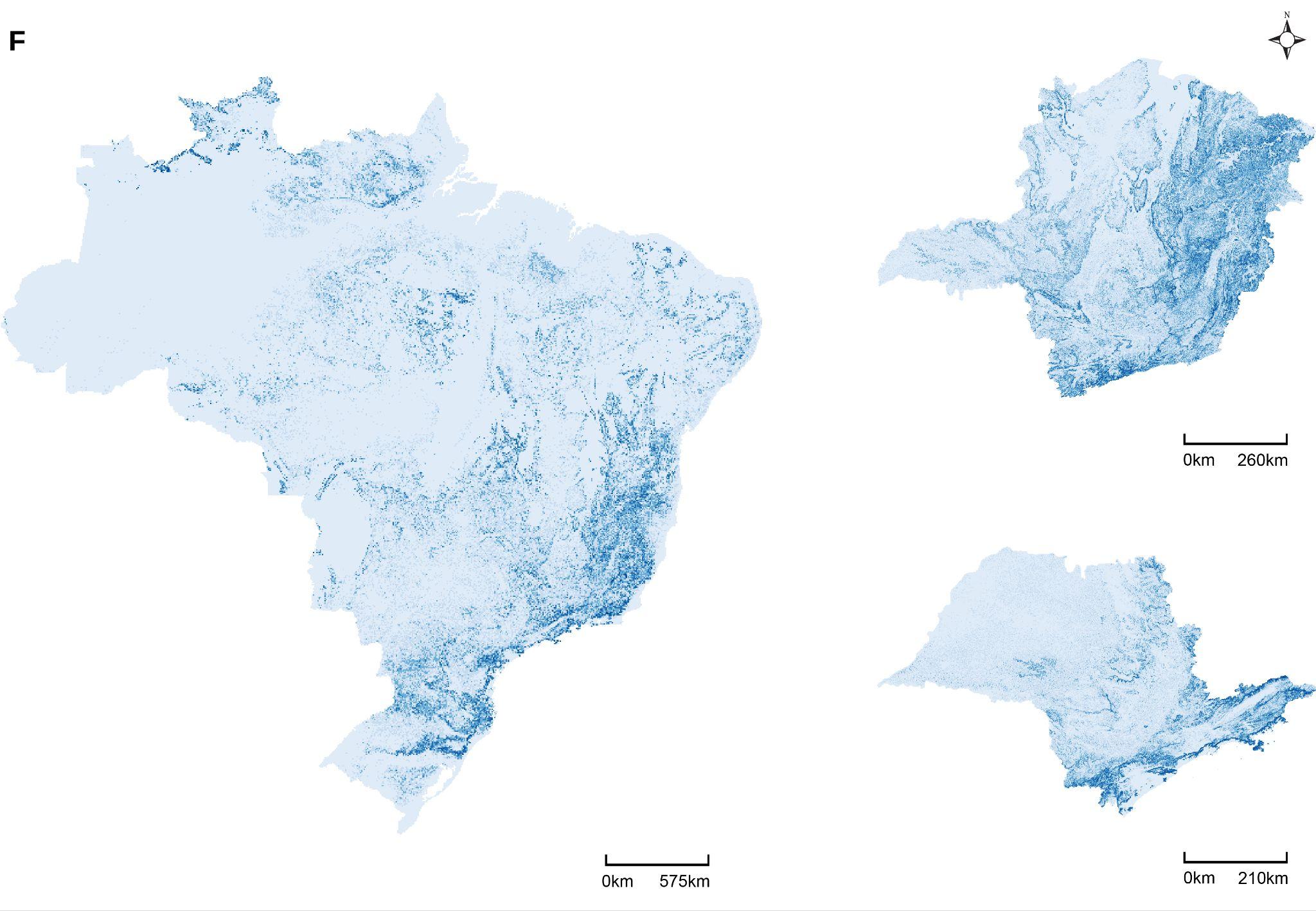

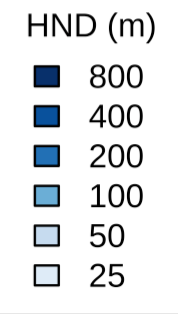


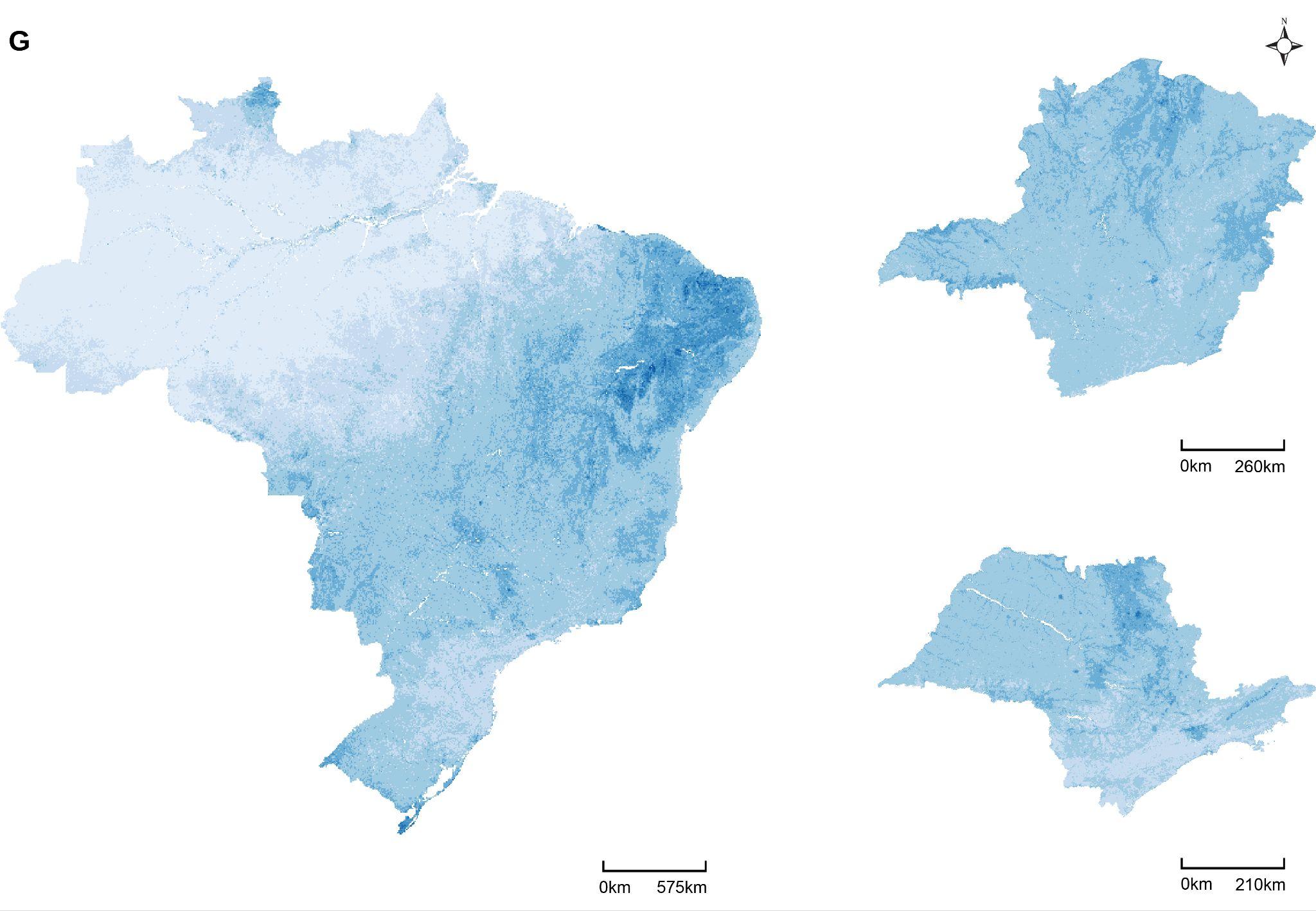

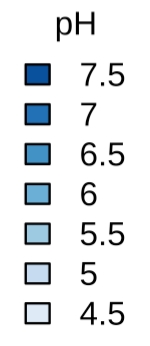


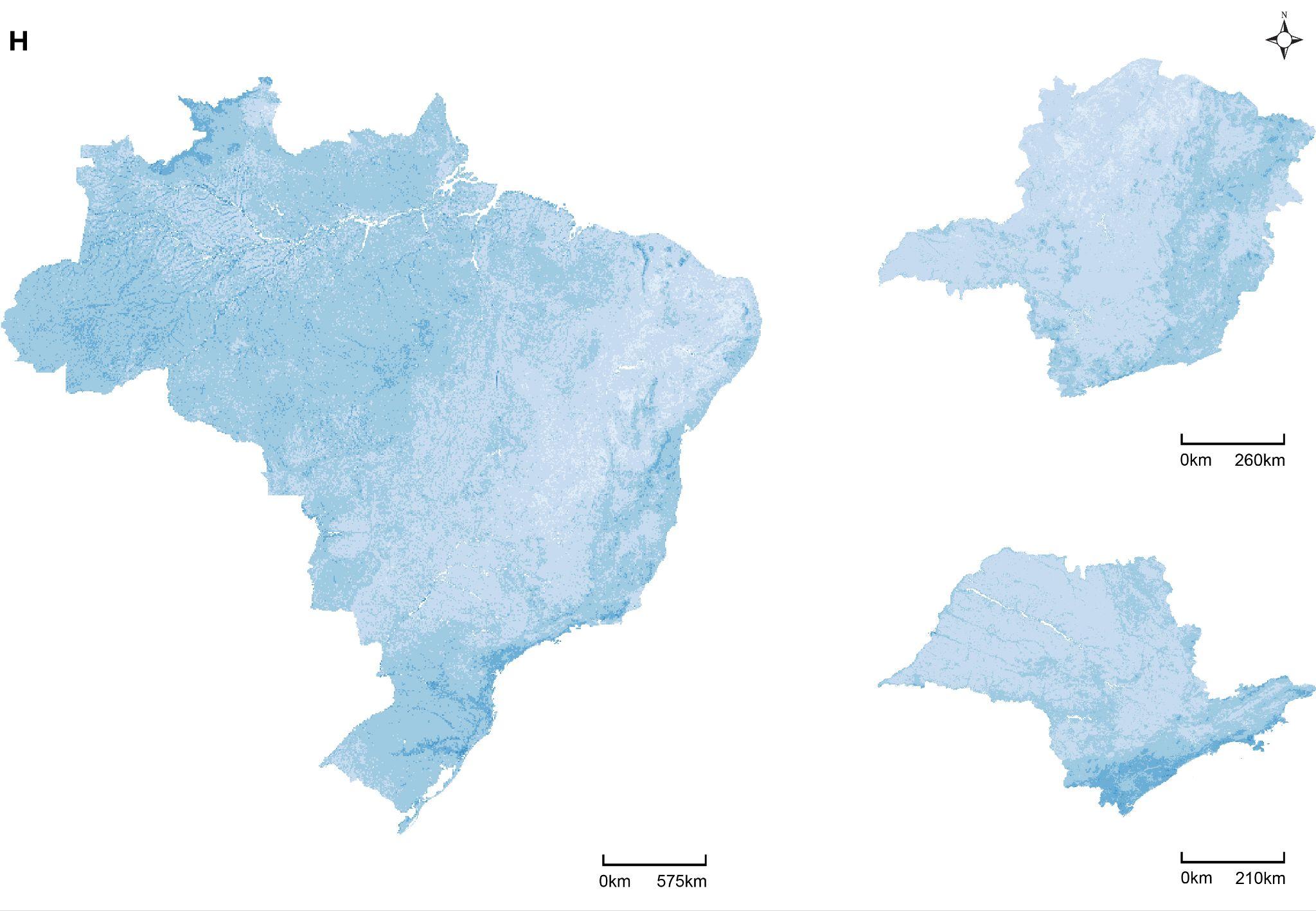

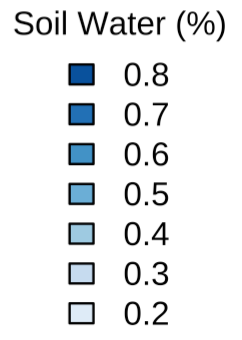


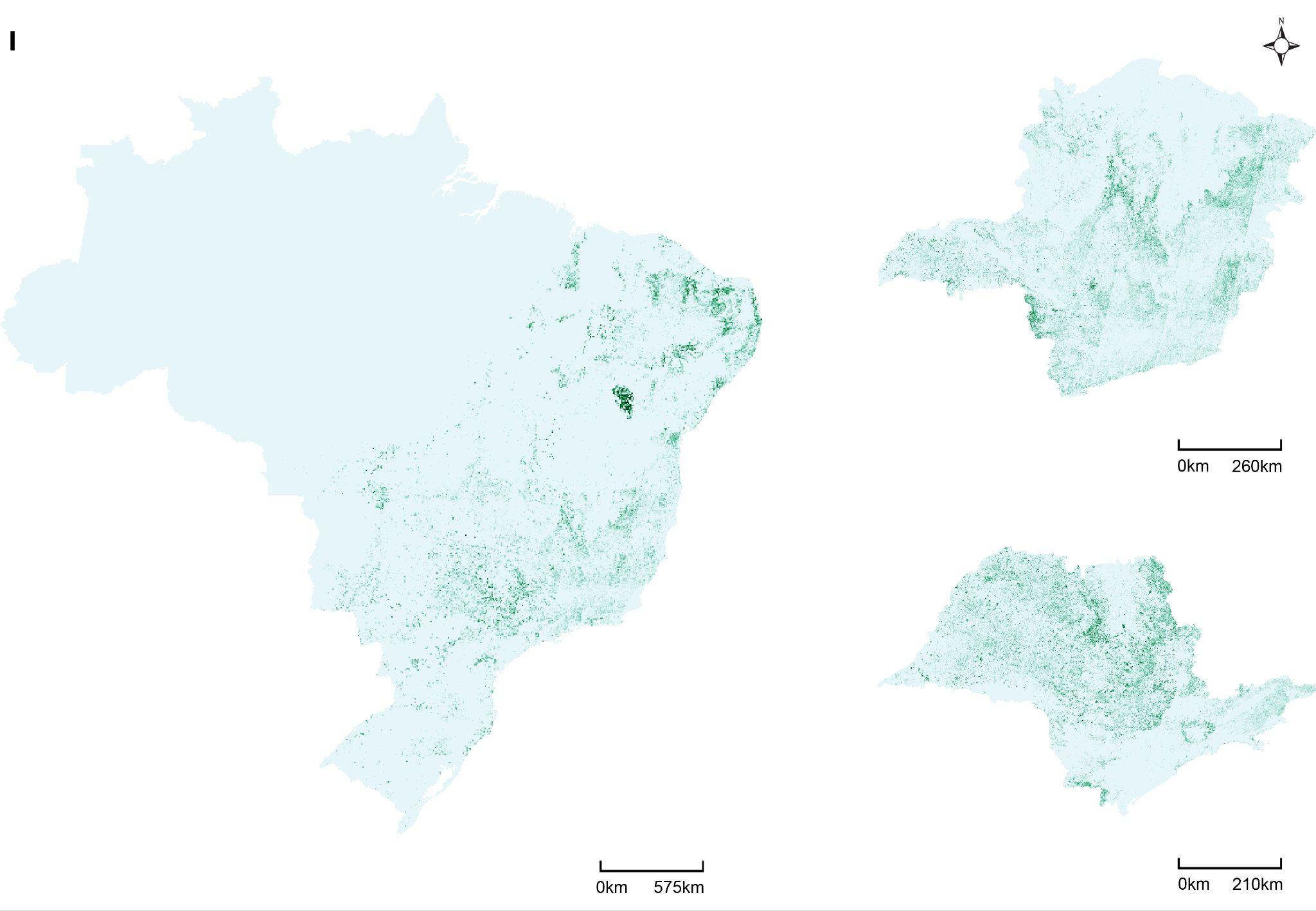

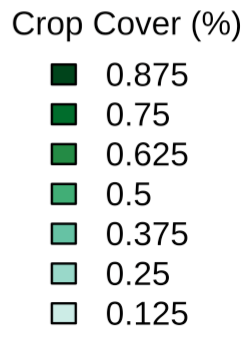


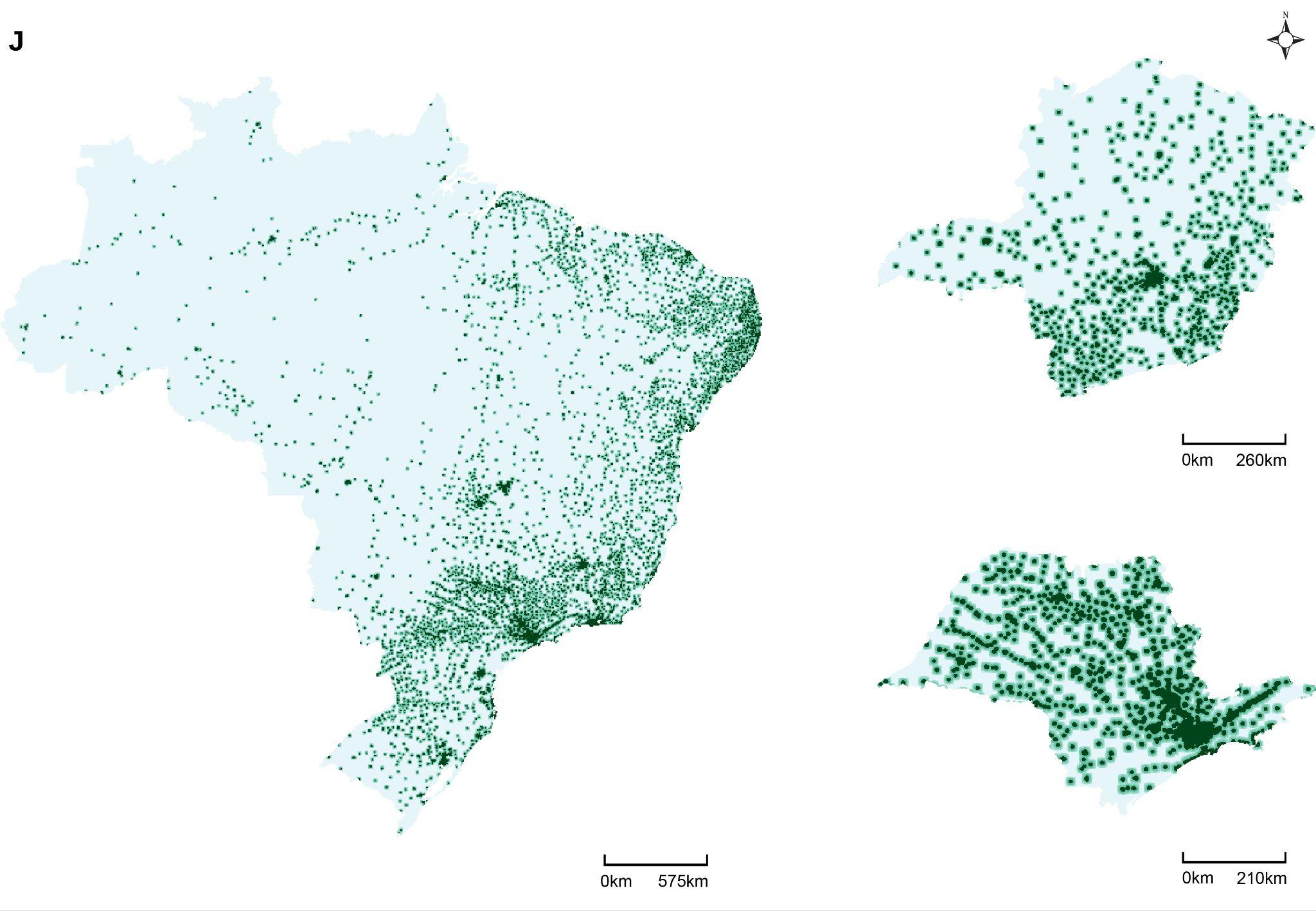

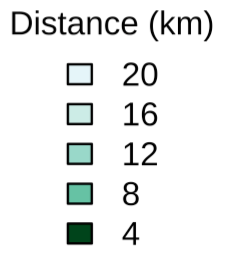


Fig B: Plots of ten-fold spatially cross-validated, out-of-sample AUC, sensitivity, specificity, pAUC, and TSS values across all three species (A, B, C), scales (panels), and model types (colors). Plots display mean (point) and standard error (error bar).

Fig C: Minas Gerais prediction maps of *B. straminea* mean suitability probabilities by model type (rows) and model geographic extent (columns). Each pixel shows the mean value across 10 bootstrapping iterations in which models were provided 80% of the available species presence records. Maps were built in R (version 4.2.2) using shapefiles from the *geobr* package [1].

Fig D: São Paulo prediction maps of *B. tenagophila* suitability probabilities by model type (rows) and model geographic extent (columns) when models were provided the full set of species presence records available. Each pixel shows the mean value across 10 bootstrapping iterations in which models were provided 80% of the available species presence records. Maps were built in R (version 4.2.2) using shapefiles from the *geobr* package [81].

Fig E: Same as Figure 6 except all models are provided the same number of data points (*n* = 115, i.e. the number of total GBIF points for *B. tenagophila* in São Paulo). Data quantity was reduced through random selection when necessary. Spatially cross-validated AUC values for *B. tenagophila* São Paulo models (all reduced to *n* = 115): expert-collected mean AUC = 0.78,+/- standard errors: [0.75, 0.80], publicly-available GBIF mean AUC = 0.79, [0.77, 0.82], and combined mean AUC = 0.76, [0.73, 0.79]. Maps were built in R (version 4.2.2) using shapefiles from the *geobr* package [81].

References

1. Pereira RHM, Gonçalves CN, et al [Internet]. 2019 [cited 2024 May 10]. geobr: Loads Shapefiles of Official Spatial Data Sets of Brazil. 2019. Available from: https://github.com/ipeaGIT/geobr
